# Supplementary material for: Human-Plant Coevolution: A modelling framework for theory-building on the origins of agriculture
Source: PLoS One. 2022 Sep 7;17(9):e0260904. doi: 10.1371/journal.pone.0260904 (PMC9451104; doi:10.1371/journal.pone.0260904)
Supplement: Attachment — Submitted filename: HPC model.docx [file pone.0260904.s002.docx]

First, it's not clear to me what this research was intended to achieve. The closest we get to this is in lines 545-7: "...we aim to show how the succession of mixed economies are intrinsic parts of coevolutionary dynamics between humans and plants, and illuminate why these culminated, in many cases, in the origins of agriculture." If this was indeed the aim then, despite claims to the contrary (see below), the paper failed to achieve this.

Rather than addressing the 'how' and 'why' questions promised in the abstract and introduction, the paper is more descriptive than analytical, nor was it clear to me how the HPC model could be used in the future to address how and why questions concerning individual cases studies or in search of some common or overarching similarities in the trajectories to agriculture in different contexts.

I therefore found the conclusions drawn towards the end of the paper unsubstantiated by the research as presented. For example: the conclusions that the HPC model “can greatly help understand…the origins of agriculture” or “produce useful insights” or “offers a solid basis for the development of generative models” – where is the evidence for this in the paper? How would the model achieve such objectives? Greater clarity of purpose and a more realistic account of achievement is needed.
